# Regional Variation in Vegetation Biomass and CSDLv2-Derived Soil C:N:P Stoichiometry Across Chinese Fir Plantations of Different Ages: An Exploratory Comparison

**DOI:** 10.3390/plants15213245

**Published:** 2026-10-22

**Authors:** Feiyang Yu, Wenfei Zhao, Yuanying Peng, Jianjun Li, Wende Yan, Xiaoyong Chen

**Affiliations:** 1 College of Computer Science and Mathematics, Central South University of Forestry and Technology, Changsha 410004, China; 20231200589@csuft.edu.cn; 2 College of Forestry, Central South University of Forestry and Technology, Changsha 410004, China; znlyzwf@csuft.edu.cn; 3 College of Arts and Sciences, Lewis University, Romeoville, IL 60446, USA; pengyu@lewisu.edu; 4 School of Low-Altitude Economy, Central South University of Forestry and Technology, Changsha 410004, China; 5 College of Water and Soil Conservation, Central South University of Forestry and Technology, Changsha 410004, China; 6 College of Arts and Sciences, Governors State University, University Park, IL 60484, USA

**Keywords:** *Cunninghamia lanceolata*, chronosequence, carbon sequestration, ecological stoichiometry, stand development

## Abstract

Regional variations in vegetation biomass and soil carbon (C), nitrogen (N) and phosphorus (P) stocks across different age classes of subtropical plantations remain incompletely characterized. This study examined variation in vegetation biomass, combined vegetation–surface–soil C stocks, soil C, N, and P stocks, and soil ecological stoichiometry across 65 Chinese fir (*Cunninghamia lanceolata* (Lamb.) Hook.) plantation plots in Hunan Province, China. The plantations were classified into five age classes: young (0–9 years), middle-aged (10–19 years), near-mature (20–24 years), mature (25–30 years), and over-mature (>30 years). Plot-level vegetation measurements were combined with spatially modeled soil properties for the upper 30 cm extracted from the China Soil Dataset for Land Surface Modeling version 2 (CSDLv2; 90 m resolution). Vegetation biomass and C stocks varied among age classes, with generally higher values in older plantations but a nonmonotonic pattern. Combined vegetation–surface–soil C stocks showed numerical variation among age classes, but the differences were not statistically significant. Soil accounted for the majority of the estimated combined C stocks, contributing approximately 76–89%, whereas vegetation contributed approximately 11–24%. Soil C, total N, and total P stocks, as well as soil C:N:P ratios, also varied numerically among age classes, but these differences were not statistically significant. These findings provide an exploratory regional comparison of vegetation biomass and C stocks alongside CSDLv2-derived soil attributes across Chinese fir plantations of different ages. Because the vegetation and soil data differ in source and spatial resolution, the combined C-stock estimates should be interpreted cautiously and do not establish age-related changes in ecosystem C partitioning or causal relationships between vegetation development and soil nutrient dynamics.

## 1. Introduction

Forest plantations play important roles in terrestrial carbon (C) sequestration, timber production, and nutrient cycling, particularly in subtropical regions where warm temperatures and abundant precipitation support high forest productivity. As plantations develop, changes in tree size, stand structure, canopy development, biomass allocation and resource availability collectively influence ecosystem C dynamics and biogeochemical processes [1,2,3]. Understanding these changes is therefore essential for evaluating the long-term ecological functions and management potential of plantation forests.

Soil biogeochemical processes also change substantially during forest development. Changes in litter production, root turnover, microbial activity and organic matter decomposition can alter the accumulation and cycling of soil C, nitrogen (N) and phosphorus (P). Because these elements are closely linked through biological demand and biogeochemical processes, their relative proportions provide useful information about nutrient availability, elemental balance and ecosystem functioning [4,5,6]. Previous studies have demonstrated that soil C:N:P stoichiometry varies with stand age, soil depth, vegetation development, and environmental conditions. In Chinese fir (*Cunninghamia lanceolata* (Lamb.) Hook.) plantations, soil C, N, and P stocks and their stoichiometric ratios can exhibit distinct age-dependent trajectories, indicating that these elements do not necessarily respond synchronously to stand development [7,8].

The relationships between aboveground development and belowground nutrient cycling are reciprocal. Increasing vegetation biomass can enhance litter production, root turnover, and rhizosphere C inputs, thereby modifying soil organic matter formation and nutrient cycling. Conversely, soil nutrient availability can constrain plant growth and influence biomass allocation and resource-acquisition strategies. Such aboveground-belowground feedback is fundamental to ecosystem development [6,9]. Recent research in Chinese fir plantations has further shown that primary productivity can regulate rhizosphere soil organic C through changes in nutrient availability and enzymatic activity, highlighting the importance of plant productivity in belowground C dynamics [10].

Chinese fir is one of the most important plantation tree species in subtropical China because of its rapid growth, high timber value, and broad geographic distribution. Chinese fir plantations occupy extensive areas of southern China and contribute substantially to regional timber production and C stock [11,12]. Previous studies have examined age-related patterns in Chinese fir biomass, C allocation, and soil nutrient stoichiometry. For example, Zhou et al. [13] reported developmental changes in tissue-specific C concentration and C distribution, whereas Tang and Wang [7] demonstrated that soil C-N-P stoichiometry varies with stand age and soil depth. Cao et al. [14] investigated age-related patterns in soil N and P stoichiometry in Chinese fir plantations and regional studies have also shown substantial spatial variation in soil C, N, and P stoichiometry across Chinese fir plantations [8]. Although previous studies have characterized age-related patterns in vegetation C allocation and soil nutrient stoichiometry, examining these properties together may help clarify how vegetation C accumulation and soil nutrient characteristics vary across Chinese fir plantation developmental stages. Hunan Province provides an appropriate regional setting for examining these relationships because Chinese fir plantations are extensively distributed throughout the province under humid subtropical climatic conditions. A regional chronosequence provides a practical space-for-time framework for assessing age-related patterns in ecosystem structure and biogeochemical properties. However, regional-scale patterns in C partitioning between vegetation and soil and soil C:N:P stoichiometry across Chinese fir plantation developmental stages remain insufficiently characterized. Using 65 plots spanning five developmental stages, this study integrates vegetation biomass and soil C and nutrient stocks estimated based on the China Soil Dataset for Land Surface Modeling version 2 (CSDLv2) to evaluate differences in ecosystem C partitioning and soil C:N:P stoichiometry among developmental stages at the regional scale. Because chronosequence studies may confound developmental-stage differences with spatial heterogeneity and other environmental factors, available environmental covariates are considered in the analyses. Nevertheless, the observational design supports the identification of associations with developmental stage rather than definitive causal effects of stand development.

In this study, we investigated age-related variation in vegetation biomass, ecosystem C stock, soil C, N, and P stocks, and soil C:N:P stoichiometry across 65 Chinese fir plantation plots representing five developmental stages in Hunan Province, China. Specifically, we aimed to (1) characterize changes in vegetation biomass and ecosystem C stock across stand developmental stages; (2) assess variation among developmental stages in soil C, N, and P stocks and their stoichiometric ratios; and (3) evaluate relationships among vegetation biomass, soil nutrient stocks, and soil C:N:P stoichiometry. We hypothesized that: (1) ecosystem C accumulation would differ among stand-development stages, with vegetation C accumulation slowing as stands mature; (2) vegetation and soil C pools would exhibit different patterns across developmental stages, resulting in variation in the relative distribution of ecosystem C between vegetation and soil; (3) soil C, N, and P would respond differently to stand development, resulting in variation in soil C:N:P stoichiometry; and (4) the strength of vegetation–soil nutrient relationships would vary among developmental stages and be associated with soil physicochemical properties.

## 2. Results

### 2.1. Age-Related Changes in Vegetation Biomass and C Stocks

**Figure 1:**
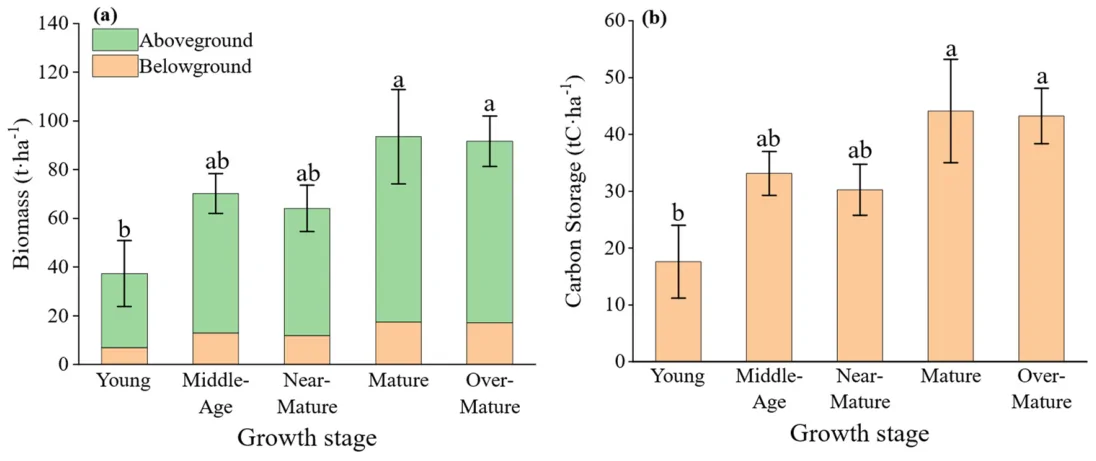
Age related changes in vegetation biomass and carbon storage across the Chinese fir plantation chronosequence. (**a**) Total vegetation biomass. (**b**) Vegetation carbon storage. Error bars represent standard deviations. Different lowercase letters indicated significant differences among developmental stages (*p* < 0.05, Tukey’s HSD test).

Vegetation biomass differed significantly among developmental stages, with generally higher biomass in older plantations (Figure 1a). Young plantations had substantially lower biomass than middle-aged and older developmental stages. Biomass accumulation was particularly pronounced during the early and intermediate stages of stand development and became comparatively slower at later stages. Vegetation C stocks showed a pattern broadly consistent with biomass accumulation (Figure 1b). Mature plantations generally contained greater vegetation C stocks than younger plantations, with higher vegetation C stocks observed across the later developmental stages.

### 2.2. Age-Related Variation in Soil Nutrient Stocks and Stoichiometry

**Figure 2:**
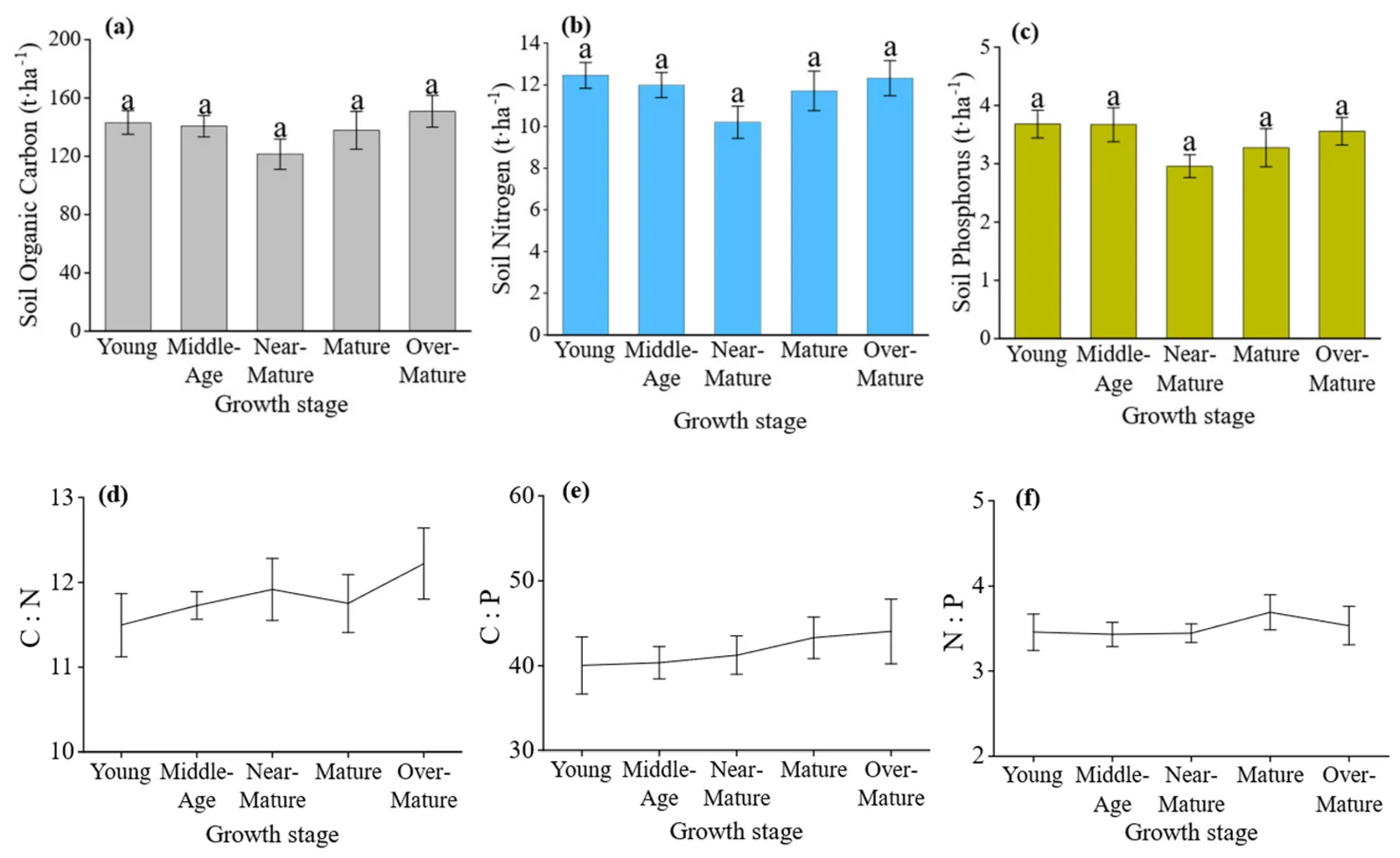
Soil nutrient stocks and ecological stoichiometric ratios across Chinese fir plantation developmental stages. (**a**) Soil organic carbon stock. (**b**) Soil total nitrogen stock. (**c**) Soil total phosphorus stock. (**d**) Soil C:N ratio. (**e**) Soil C:P ration. (**f**) Soil N:P ratio. Error bars represent standard deviations. Different lowercase letters indicate significant differences among developmental stages (*p* < 0.05, Tukey’s HSD test).

Soil C, N, and P stocks exhibited distinct patterns across the chronosequence (Figure 2). Soil organic carbon (SOC) stocks generally increased with stand age (Figure 2a). Soil TN stocks also varied among developmental stages and were generally greater in older plantations (Figure 2b). In contrast, soil TP stocks exhibited comparatively different age-related dynamics (Figure 2c), with P accumulation not closely paralleling the patterns observed for C and N. The contrasting patterns of soil C, N, and P were associated with variation in soil C:N, C:P, and N:P ratios across stand-development stages (Figure 2d–f).

### 2.3. Changes in Combined Vegetation–Surface–Soil C Stocks and C Partitioning

**Figure 3:**
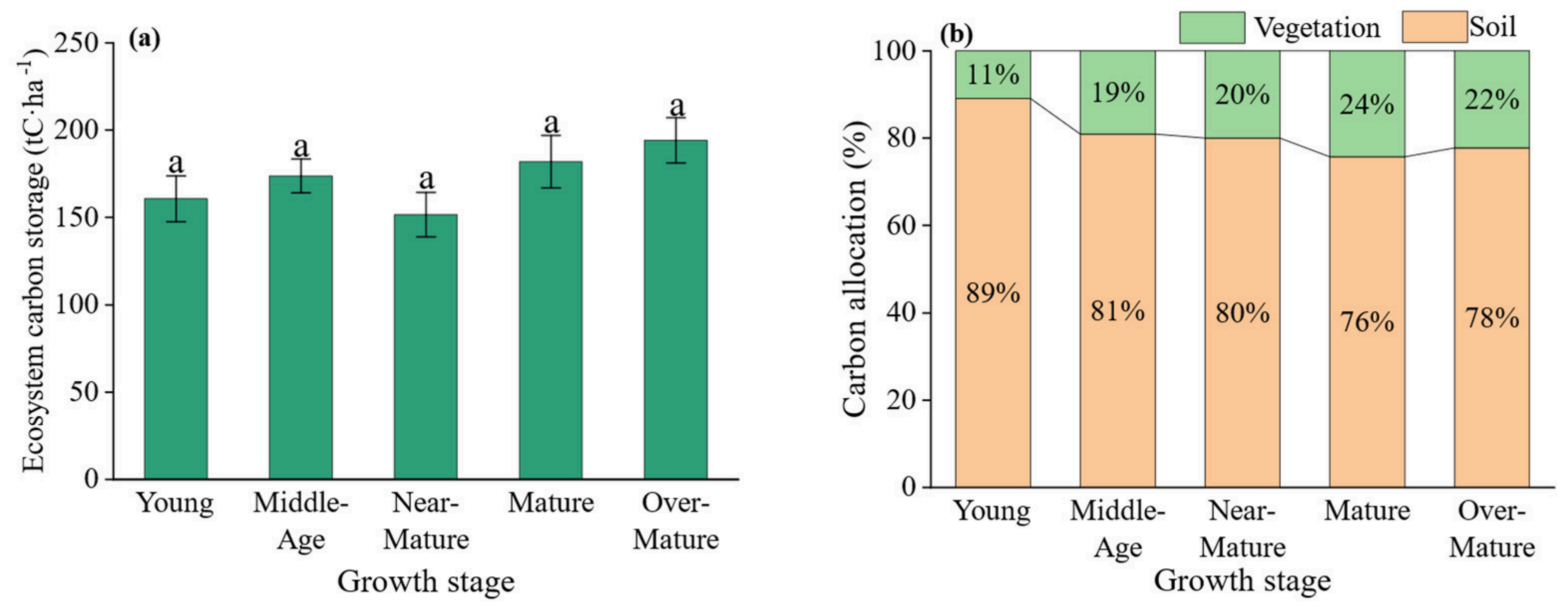
Changes in combined vegetation–surface–soil C stocks and C partitioning across the Chinese fir chronosequence. (**a**) Combined C stock calculated as the sum of vegetation C and SOC in the 0–30 cm soil layer., with vegetation and soil components shown separately. (**b**) Relative proportions of vegetation and surface-soil C to the combined C pool within each developmental stages. Error bars represent standard deviations. Different lowercase letters indicate significant differences between developmental stages (*p* < 0.05, Tukey’s HSD test).

The combined vegetation–surface–soil C pool increased across the chronosequence (Figure 3a), with higher C stocks in vegetation and the upper 30 cm of soil observed across the developmental stages. Older plantations generally contained greater combined C stocks than younger plantations.

Importantly, soil remained the dominant C pool within the combined vegetation–surface–soil pool throughout stand development. In young plantations, soil C accounted for approximately 89% of the combined pool, whereas vegetation C accounted for approximately 11% (Figure 3b). The contribution of vegetation C increased to approximately 19% in middle-aged stands and 20% in near-mature stands. In mature and over-mature plantations, vegetation C accounted for approximately 24% and 22%, respectively. The relative contribution of vegetation C was greater in mature and over-mature plantations, whereas soil C in the upper 30 cm remained the larger component of the quantified C pool.

### 2.4. Relationships Among Vegetation Biomass, Soil Properties and Nutrient Stoichiometry

**Figure 4:**
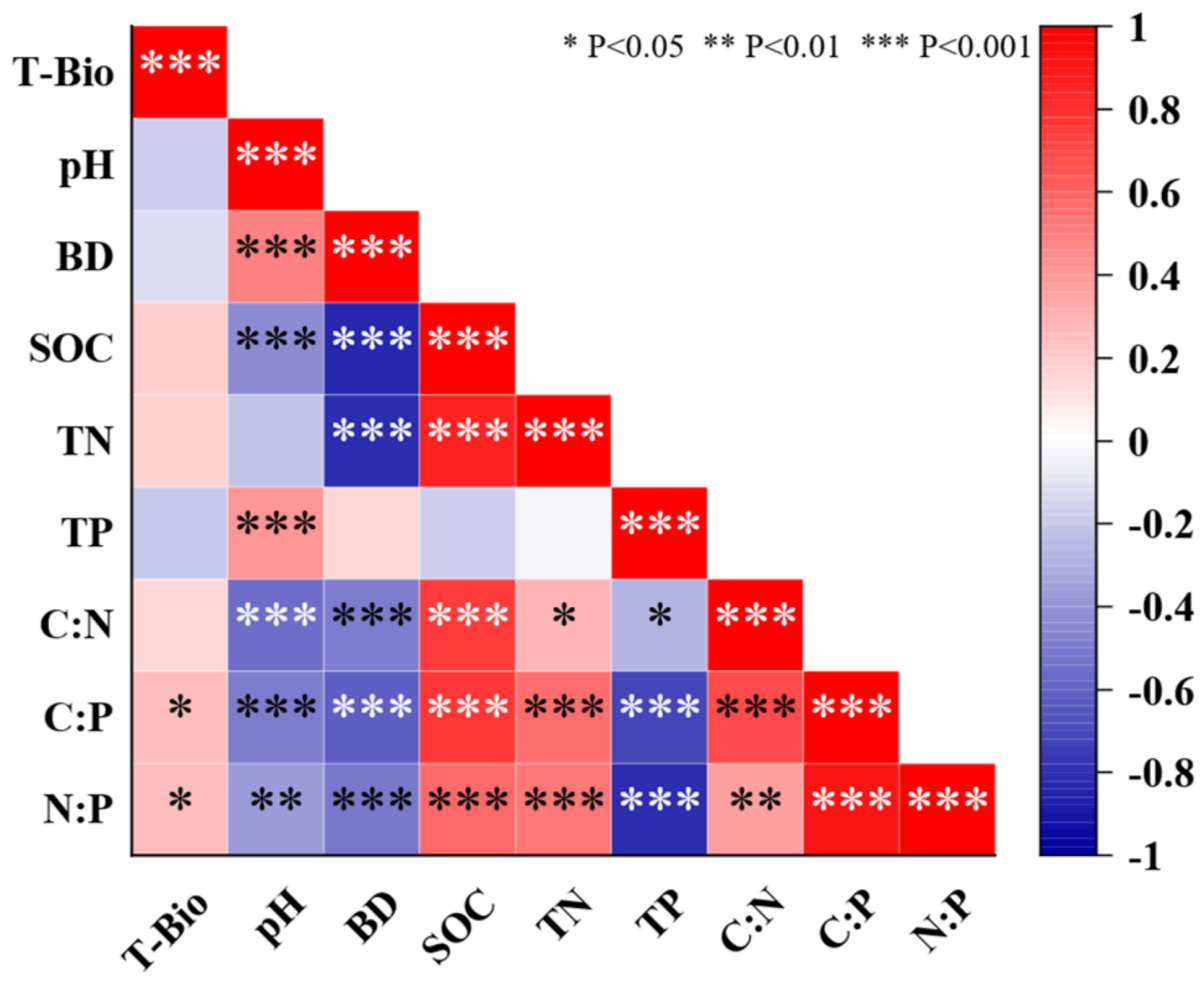
Correlation matrix shows relationships among vegetation biomass, soil properties, nutrient stocks and stoichiometric characteristics. Color intensity represents correlation coefficients. T-Bio: total biomass; SOC: soil organic carbon; TN: total nitrogen; TP: total phosphorus; BD: bulk density.

Correlation analyses revealed significant associations among vegetation biomass, soil nutrient stocks, and ecological stoichiometric variables (Figure 4). Total vegetation biomass was positively associated with soil C, N, and P stocks. SOC was also strongly associated with TN and TP. Soil pH and bulk density were significantly related to several nutrient stocks and stoichiometric ratios, with significant associations observed between soil physicochemical properties and nutrient pools and elemental ratios across the study sites.

## 3. Discussion

### 3.1. Stand Development Promotes Biomass Accumulation and Ecosystem C Stock

Vegetation biomass and C stocks increased with stand age, supporting the first hypothesis and showing an association between stand-development stage and vegetation C accumulation in Chinese fir plantations (Figure 1). The increase in vegetation C likely reflects progressive increases in tree size and woody biomass as stands develop. Previous studies have reported substantial age-related changes in biomass and C stocks in Chinese fir plantations and other forest ecosystems [1,3,11,13,14]. The observed variation in biomass across developmental stages is consistent with general patterns of forest stand development. During early establishment, trees rapidly occupy available growing space and increase canopy and stem biomass. As competition intensifies, self-thinning and resource limitation can constrain stand-level biomass accumulation. Chinese fir growth and biomass production are also influenced by initial planting density and associated competition [11]. At later developmental stages, declining relative growth rates and increasing allocation to structural tissues may be associated with slower net biomass accumulation.

The present findings are consistent with evidence that Chinese fir exhibits substantial age-related changes in C concentration, C stock, and C distribution among tissues [13]. Recent research further demonstrates that forest age, tree size, and climate jointly are associated with variation in biomass and C-storage allocation in Chinese fir forests [3]. Thus, the observed increase in vegetation C with stand age should be viewed as an integrated pattern associated with differences in stand structure and development, rather than as a simple linear effect of chronological age.

The increase in quantified partial ecosystem C pool, comprising vegetation C and soil C within the upper 30 cm, was associated with higher C stocks in these components across developmental stages. This quantified C pool does not include deeper soil C, roots, deadwood, litter, or other ecosystem C pools that were not quantified in this study. Therefore, it should not be interpreted as representing total ecosystem C storage. Standing C stocks should also not be equated directly with annual C sequestration rates.

Net ecosystem C sequestration depends on the balance among photosynthetic C uptake, autotrophic and heterotrophic respiration, biomass mortality, decomposition, and C transfer into persistent soil pools. Consequently, longer rotations may increase standing C storage, but their effects on annual sequestration rates and long-term C permanence require additional measurements.

### 3.2. Stand Age Modifies Soil C:N:P Stoichiometry Through Divergent Nutrient Dynamics

Soil C and N stocks generally increased with stand age, whereas P exhibited weaker or more variable age-related changes. This divergence was associated with variation in soil C:N:P stoichiometry (Figure 2). Such contrasting responses are consistent with the concept that C, N, and P are regulated by partially distinct biological and geochemical processes [4,5,15].

The increase in SOC with stand age may be associated with increasing inputs of litter, roots, and other plant-derived organic materials as vegetation develops. Plant productivity and belowground C inputs can influence SOC through microbial processing and rhizosphere interactions. In Chinese fir plantations, primary productivity has been shown to regulate rhizosphere SOC through effects on nutrient availability and enzymatic activities [10]. Recent research also indicates that microbial residue C can represent an important component of SOC sequestration in Chinese fir plantations, providing a potential pathway linking plant-derived inputs to longer-term soil C accumulation [16]. The increase in soil N stocks with stand age may similarly be associated with greater organic matter inputs and enhanced retention of N within soil organic matter. However, the mechanisms underlying N accumulation cannot be directly established from the present dataset because microbial processes, mineral N pools, biological N fixation, and N mineralization were not measured.

In contrast, soil P dynamics were less closely coupled to stand age than C and N. Phosphorus cycling differs from N cycling because P is strongly influenced by parent material, mineral weathering, adsorption and desorption, and biological recycling [15]. The relative stability of soil P alongside increasing C and N was associated with variation in C:P and N:P ratios during stand development. Importantly, changes in C:P and N:P ratios provide evidence of changing elemental balance but do not, by themselves, demonstrate P limitation. Nutrient limitation should ideally be evaluated using measurements of plant tissue stoichiometry, nutrient resorption, soil available P, phosphatase activity, nutrient-addition experiments, or other physiological indicators. Previous Chinese fir research also indicates that stand-age effects on soil C-N-P stoichiometry can be nonlinear and dependent on soil depth [7]. Regional evidence further demonstrates that Chinese fir soil stoichiometry is strongly influenced by geographical and climatic variables [8].

### 3.3. C Partitioning Shifts During Plantation Development

Although soil C in the upper 30 cm remained the largest component of the quantified vegetation–surface–soil C pool throughout stand development, the relative contribution of vegetation C increased from approximately 11% in young plantations to 22–24% in mature and over-mature plantations (Figure 3). This pattern shows variation in the distribution of quantified C between vegetation and soil across developmental stages. The observed pattern is consistent with changes in C allocation during forest development. Young trees allocate C among roots, leaves, stems and other tissues required for establishment and resource acquisition. As stands develop, increasing tree size and structural biomass may be associated with greater C storage in long-lived woody tissues [17]. Similar differences in the relative distribution of plant and soil C have been documented among forest developmental stages [18]. Nevertheless, the increase in the vegetation contribution should not be interpreted as evidence that soil C becomes unimportant in older plantations. Soil continued to represent the largest ecosystem C pool at every developmental stage examined here. This finding emphasizes that effective forest C management should address both biomass C and soil C.

Recent evidence further suggests that the mechanisms responsible for SOC stabilization can differ among soil types. In Chinese fir plantations, red and yellow soils appear to differ in the relative importance of geochemical preservation and physical protection in SOC stabilization [19]. Consequently, strategies aimed at increasing long-term C storage should consider not only stand age and biomass accumulation but also site-specific soil characteristics and C-stabilization mechanisms.

### 3.4. Coupling Between Vegetation Development and Soil Nutrient Dynamics

The correlations between vegetation biomass and soil C, N, and P stocks did not demonstrate statistically significant relationships (Figure 4). Such relationships are ecologically plausible because increasing vegetation biomass can enhance litter inputs, root turnover, and rhizosphere C inputs, whereas soil nutrient availability can influence plant growth and resource acquisition [6,9]. Although SOC, TN, and TP showed variation among plots, the correlations did not consistently demonstrate significant associations among all three soil nutrient pools. However, these correlations should not be interpreted as evidence of direct causal relationships [20]. Both vegetation and soil properties may respond simultaneously to common environmental drivers such as climate, topography, soil type and stand-management history.

Soil pH and bulk density were also associated with nutrient stocks and stoichiometric ratios [21]. Soil pH can influence nutrient availability, microbial activity, and mineral interactions, whereas bulk density is related to soil porosity, aeration, water movement, and the physical protection of organic matter. These properties represent important environmental factors associated with variation in soil nutrient pools and stoichiometric relationships. However, correlations involving soil stocks and stoichiometric ratios should be interpreted cautiously because bulk density contributes to the calculation of soil stocks and the ratios are derived from the underlying elemental measurements. These shared components can introduce mathematical dependencies, so the observed correlations do not necessarily represent independent ecological relationships. The observed relationships are consistent with evidence that productivity and belowground C processes are closely linked in Chinese fir plantations. Chen et al. [10] demonstrated that primary productivity can regulate rhizosphere SOC through effects on nutrient availability and enzyme activities. However, age-related patterns may not always be monotonic, emphasizing the importance of considering developmental stage rather than assuming a simple linear relationship between productivity and soil C.

### 3.5. Implications for Plantation Management

As stand age increases, vegetation and ecosystem C stocks rise, with high levels of stand C stock observed across all developmental stages, suggesting that maintaining plantations through sufficiently long developmental periods may help achieve higher levels of stand C sequestration. However, decisions regarding rotation length should consider multiple ecosystem functions rather than C stocks alone [22]. In particular, the divergence between C and N accumulation and the comparatively limited variation in P suggests that nutrient availability may become increasingly important for sustaining productivity as plantations develop. Nevertheless, the present study did not directly measure plant nutrient limitation or soil available P and therefore cannot establish that older plantations are P-limited.

Management practices that minimize unnecessary soil disturbance, maintain litter inputs, protect soil structure, and sustain nutrient cycling may help preserve both soil C and soil fertility. Because soil C stabilization pathways can vary among soil types [19], management strategies should also account for site-specific soil characteristics. Thus, optimizing Chinese fir plantation management should consider the joint dynamics of stand development, biomass accumulation, soil C stock, and nutrient stoichiometry rather than focusing exclusively on timber production or aboveground C stocks [8].

### 3.6. Limitations and Future Research

Several limitations should be considered when interpreting the present findings.

First, soil properties were derived from the CSDLv2 spatial dataset rather than direct field measurements. Although CSDLv2 provides high-resolution spatial predictions of multiple soil properties, modeled soil properties inevitably contain prediction uncertainty that may influence estimates of nutrient stocks and stoichiometric relationships [23]. Future studies should validate database-derived soil properties with field measurements. In addition, the CSDLv2-derived values represent spatial model estimates at a 90 m resolution and therefore should not be interpreted as direct measurements of soil conditions at the plantation plots. Second, the analysis was restricted to the 0–30 cm soil layer. Deeper soil horizons can contain substantial amounts of organic C and nutrients, and their responses to stand development may differ from those of surface soils. Future research should therefore examine deeper soil profiles.

Third, the chronosequence approach represents a space-for-time substitution. Differences among stands of different ages may therefore reflect not only stand development but also variation in climate, soil type, topography, stand history, and management practices. Although climatic and topographic covariates were incorporated into the analyses, residual environmental and management effects cannot be completely excluded. Moreover, the vegetation and soil datasets were not collected under an identical field-sampling framework. Vegetation biomass and C estimates were associated with plantation-plot data, whereas soil C, N, and P variables were obtained from the spatially modeled CSDLv2 dataset. Thus, the combined ecosystem C estimates integrate plot-associated vegetation data with modeled soil estimates and should be interpreted accordingly.

Fourth, the study did not directly measure microbial communities, extracellular enzyme activities, available nutrient pools, litter production, root turnover, or SOC fractions. Consequently, mechanisms underlying the observed changes in soil C:N:P stoichiometry and C accumulation remain inferential. Future research combining chronosequence surveys with measurements of microbial communities, extracellular enzyme activities, particulate and mineral-associated organic C, and nutrient availability would provide stronger mechanistic evidence. Finally, the present study is observational. The observed correlations between vegetation biomass and soil nutrient stocks represent statistical associations but do not establish causal pathways. Long-term monitoring and manipulative experiments, including nutrient-addition and thinning experiments, would be valuable for determining how stand development and nutrient availability interact to regulate C sequestration.

A key limitation of this study is that vegetation attributes and soil properties were derived from different data sources and therefore were not collected under an identical field-sampling framework. Vegetation biomass and C estimates were associated with plantation-plot data, whereas soil C, N, and P variables were obtained from the CSDLv2 spatially modeled dataset. Consequently, the CSDLv2-derived soil values should be interpreted as spatial estimates rather than direct measurements of soil conditions at the plantation plots. Because CSDLv2 provides spatially modeled soil-property estimates rather than repeated measurements over time, it may not capture soil changes specifically associated with stand development. Therefore, the CSDLv2 data cannot independently establish temporal changes in soil properties as plantations age. In addition, the regional space-for-time comparison among stands of different ages cannot separate the influence of stand age from differences in climate, topography, soil environment, geographic location, and other site-specific factors. Therefore, the observed differences in soil C, N, P, and C:N:P stoichiometry should be interpreted as comparative spatial patterns associated with forests at different developmental stages rather than as definitive causal effects of stand development. Field-based studies using co-located vegetation and soil sampling across well-matched chronosequences are needed to directly test these relationships.

## 4. Materials and Methods

### 4.1. Study Site

**Figure 5:**
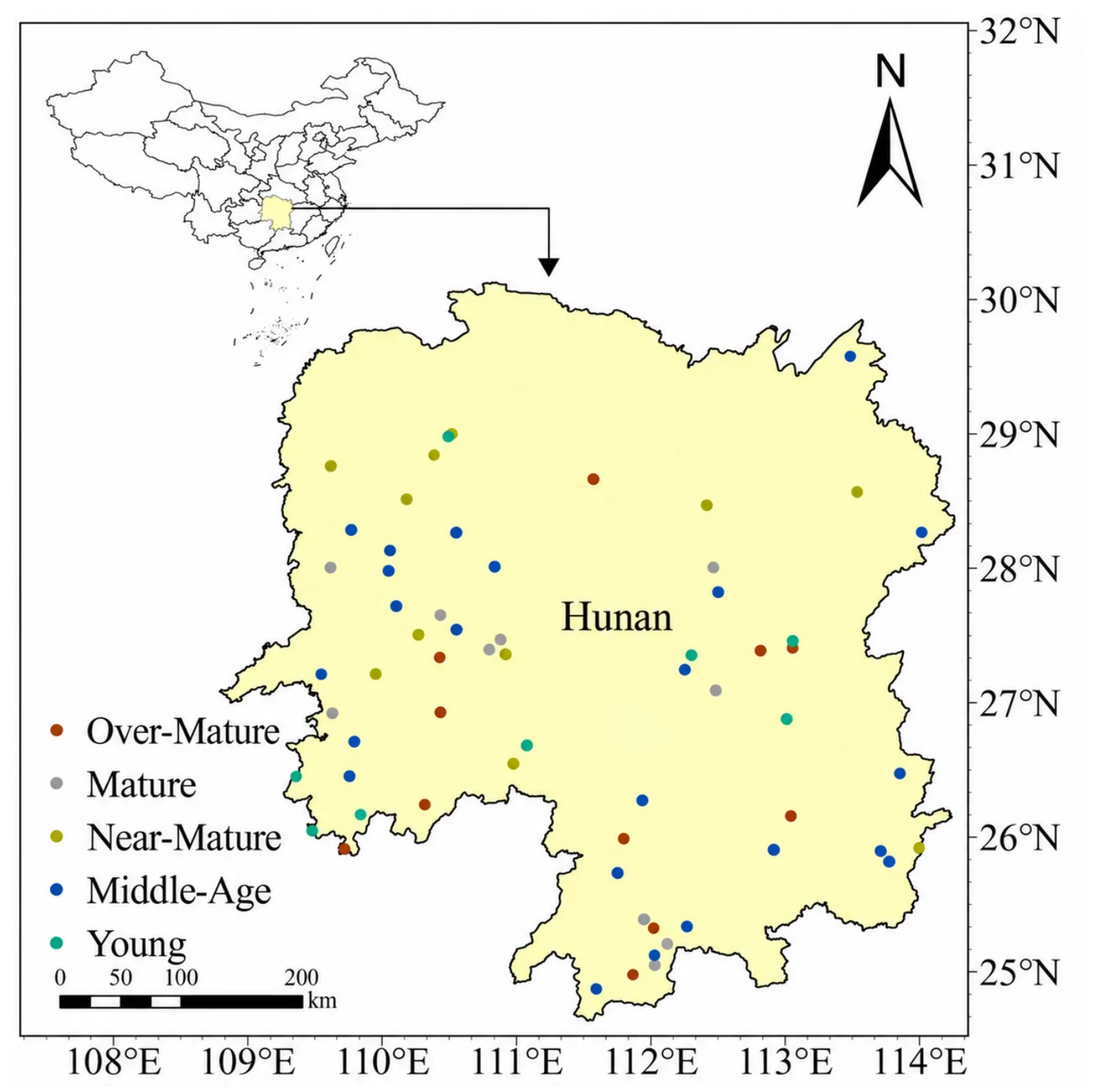
Spatial distribution of the 65 Chinese fir plantation plots across Hunan Province, subtropical China. Colored symbols represent five developmental stages: young (0–9 years), middle-age (10–19 years), near-mature (20–24 years), mature (25–30 years), and over-mature (>30 years). The inset shows the location of Hunan Province in China.

This study was conducted across Hunan Province in south-central China (108°47′−114°15′ E, 24°38′−30°08′ N; Figure 5). Hunan is in the middle reaches of the Yangtze River Basin and is characterized predominantly by mountainous and hilly terrain, including the Wuling, Xuefeng, Luoxiao, and Nanling mountain systems. The region has a humid subtropical monsoon climate with distinct seasonal variation. Mean annual temperature generally ranges from 16 to 18 °C, and mean annual precipitation is approximately 1450 mm. The combination of warm temperatures and abundant precipitation provides favorable conditions for subtropical forests, including extensive Chinese fir plantations.

### 4.2. Sampling Design and Chronosequence

A regional-scale observational design was used to characterize age-related patterns associated with stand-development stage in Chinese fir plantations (Figure 5). A total of 65 Chinese fir plantation plots were selected across Hunan Province to represent a broad range of stand developmental stages and geographic conditions. The selected plots encompassed variation in climate, topography, and soil conditions. Because the study used a regional space-for-time substitution, the plots represented different stands rather than repeated observations of the same stands through time.

Based on stand age, plantations were classified into five developmental stages: young (0–9 years), middle-aged (10–19 years), near-mature (20–24 years), mature (25–30 years), and over-mature (>30 years). The numbers of plots in the five developmental stages were 9, 23, 11, 10 and 12, respectively. Each sampling plot had a size of 667 m^2^ (Table S1). These developmental stages were used as a comparative framework for examining patterns in vegetation biomass, ecosystem C stock, soil nutrient stocks, and soil C:N:P stoichiometry across the regional stand-development gradient. The observational design does not allow associations with stand-development stage to be fully disentangled from geographic, environmental, or management differences among sites. To account for environmental heterogeneity, mean annual temperature, mean annual precipitation, elevation, slope and aspect were included as covariates in the statistical analyses, where appropriate. Because this study employed a space-for-time substitution, environmental heterogeneity among sites was considered in the statistical analyses. Mean annual temperature, mean annual precipitation, elevation, slope and aspect were included as covariates where appropriate.

### 4.3. Vegetation Biomass and C Stocks

Vegetation biomass and C-stocks were estimated from individual-tree diameter at breast height (DBH, D; cm) measurements from the 65 Chinese fir plantation plots. Aboveground biomass (W_AGB_, kg tree^−1^) and belowground biomass (W_BGB_, kg tree^−1^) of individual trees were estimated using species-specific allometric equations [24]:

W_AGB_ = 0.07637 × D^2^·^40393^ (1)

W_BGB_ = 0.015504 × D^2^·^44421^ (2)

Individual-tree aboveground C stocks (C_AGC_, kg C tree^−1^) and belowground C stocks (C_BGC_, kg C tree^−1^) were calculated by multiplying the corresponding biomass estimates by C-concentration coefficients of 0.4786 and 0.4408, respectively [25]:

C_ABC_ = W_AGB_ × 0.4786 (3)

C_BGC_ = W_BGB_ × 0.4408 (4)

Individual-tree biomass and C stocks were summed across all measured Chinese fir trees within each plot to obtain plot-level aboveground, belowground, and total values. Total biomass and total vegetation C stock were calculated as the sums of their aboveground and belowground components. Each plantation plot had a fixed area of 667 m^2^. Plot-level biomass and C-stock totals, expressed in kg plot^−1^ and kg C plot^−1^, respectively, were converted to area-based values (t ha^−1^ and t C ha^−1^).

The resulting plot-level vegetation biomass and C-stock estimates are provided in a supplementary table (Table S2). Total ecosystem C storage is calculated as the sum of vegetation C storage and soil organic C storage (for the 0–30 cm soil depth) estimated based on CSDLv2.Vegetation attributes and soil properties were not derived from an identical field-sampling framework. Vegetation biomass and C estimates were associated with the plantation plots, whereas soil properties were obtained from spatially modeled CSDLv2 data extracted at the corresponding plot coordinates. Therefore, the resulting ecosystem C stock represents an integrated estimate based on plot-associated vegetation data and spatially modeled soil data.

### 4.4. Soil Data Acquisition and Processing

Soil property data were obtained from the China Soil Dataset for Land Surface Modeling version 2 (CSDLv2; https://doi.org/10.11888/Terre.tpdc.301235), released by the National Tibetan Plateau Data Center. CSDLv2 is a spatially explicit, model-derived soil database developed using a spatially explicit model-derived soil database developed using harmonized soil-profile observations, environmental covariates, and machine-learning methods [23]. CSDLv2 provides predicted soil-property maps at a 90 m spatial resolution for six standard depth intervals: 0–5, 5–15, 15–30, 30–60, 60–100 and 100–200 cm [17]. The variables used in this study included soil bulk density (BD), soil organic C (SOC), total nitrogen (TN), total phosphorus (TP), and soil pH.

The geographic coordinates of the 65 plantation plots were imported into ArcGIS Pro 2.8 (Esri, Redlands, CA, USA), and corresponding soil-property values were extracted from the CSDLv2 raster layers using the Extract Multi Values to Points tool. The raster coordinate system was GCS_WGS_1984, and values were extracted at the plot coordinates without applying a buffer or bilinear interpolation. Because CSDLv2 provides modeled estimates at a 90 m spatial resolution rather than direct field measurements, the extracted values were interpreted as estimates of soil conditions at the plot locations. The 0–30 cm soil profile was represented by three CSDLv2 depth intervals: 0–5, 5–15 and 15–30 cm. Soil properties for the 0–30 cm profile were calculated by integrating the estimates across these three intervals rather than treating the profile as a single directly mapped layer.

The original dataset stored soil-property values as integers. Stored values for the soil properties used in this study were divided by 100 to recover their actual values. The original missing-value code (−32768) and empty extraction results were treated as invalid data, and records with missing values for variables required in each analysis were excluded. SOC, TN, and TP represented the principal soil C, N, and P pools, respectively. Bulk density was used to convert nutrient concentrations into area-based stocks, whereas soil pH was considered an indicator of soil chemical conditions that may influence nutrient availability and biogeochemical processes.

### 4.5. Calculation of Soil Nutrient Stocks and Stoichiometric Ratios

Soil C, N, and P stocks were calculated from nutrient concentration, bulk density, and soil-layer thickness according to

Stock = C × BD × D × 0.1(5)

where Stock is the soil nutrient stock (Mg ha^−1^), C is the nutrient concentration (g kg^−1^), BD is soil bulk density (g cm^−3^), and D is soil-layer thickness (cm). The conversion factor of 0.1 accounts for the conversion of the concentration, bulk-density, soil-layer thickness, and area units to t ha^−1^. For the 0–30 cm profile, nutrient stocks were calculated separately the 0–5, 5–15, and 15–30 cm layers and then summed

Stock_0–30_ = Stock_0–5_ + Stock_5–10_ + Stock_15–30_ (6)

Soil C:N, C:P, and N:P ratios were calculated from elemental concentrations expressed on consistent mass units. Because all ratios represent elemental stoichiometry, the numerator and denominator were converted to compatible elemental units before calculation. These ratios were calculated from the CSDLv2-derived soil-property estimates and therefore represent modeled spatial estimates of soil elemental stoichiometry.

### 4.6. Statistical Analysis

All statistical analyses were conducted using R version 4.2.0 [26]. Means and standard deviations were calculated for vegetation biomass, vegetation and ecosystem C stocks, soil properties, nutrient stocks, and stoichiometric ratios within each stand-developmental stage. Data distributions were evaluated using the Shapiro–Wilk test, and homogeneity of variance was assessed using Levene’s test. Variables were transformed when necessary to satisfy the assumptions of parametric analyses.

One-way analysis of variance (ANOVA) was used to compare variables among the five stand-development classes. When significant differences were detected (*p* < 0.05), Tukey’s honestly significant difference (HSD) test was used for pairwise comparisons.

Because the regional space-for-time comparison involved environmental heterogeneity among sampling sites, analysis of covariance (ANCOVA) was used where appropriate to assess whether differences among stand-development classes remained after accounting for selected climatic and topographic covariates. These covariates were included to account for measured environmental variation but cannot fully account for unmeasured site characteristics, geographic variation, or environmental information incorporated into the CSDLv2 modeling framework. Accordingly, the adjusted results were interpreted as associations rather than definitive causal effects of stand development.

Pearson correlation analysis was used to examine relationships among vegetation biomass, CSDLv2-derived soil properties, nutrient stocks, and stoichiometric ratios. Spearman’s rank correlation was used when the assumptions for Pearson correlation were not satisfied. All statistical tests were two-sided, and statistical significance was evaluated at α = 0.05.

## 5. Conclusions

Across 65 Chinese fir plantation plots in Hunan Province, vegetation biomass and C stocks varied among five plantation age classes, with generally higher values in older classes but a nonmonotonic pattern. Combined vegetation–surface–soil C stocks also varied numerically among age classes, but the differences were not statistically significant. Soil represented the larger estimated component of the combined quantified C stocks, accounting for approximately 76–89%, whereas vegetation accounted for approximately 11–24%. Soil C, total N, and total P stocks, as well as soil C:N:P stoichiometry ratios, showed numerical variation among age classes, but the differences were not statistically significant.

These findings provide an exploratory regional comparison of vegetation biomass and C stocks alongside modeled soil C, N, and P attributes across Chinese fir plantations of different ages. However, vegetation measurements and CSDLv2-derived soil properties differ in source and spatial resolution, and the observational space-for-time substitution design cannot fully separate stand age from environmental variation. Accordingly, the observed patterns do not establish causal effects of stand development, definitive changes in ecosystem C partitioning, or age-related shifts in soil nutrient limitation. Considering vegetation characteristics together with soil ecological stoichiometry may provide a useful comparative framework for examining C–nutrient relationships and informing future assessments of Chinese fir plantation management [27]. Further studies using spatially matched, plot-level vegetation and soil measurements, together with direct assessments of soil nutrient availability, are needed to evaluate these relationships and their implications for plantation management.

## Data Availability

The original contributions presented in this study are included in the article. Because the study uses third-party spatial soil data, the availability and redistribution conditions of the underlying CSDLv2 dataset should be stated separately where applicable. Further inquiries can be directed to the corresponding authors.

## Supplementary Materials

The following supporting information can be downloaded at https://www.mdpi.com/article/10.3390/plants15213245/s1: Table S1. Original basic information of 65 plots of Chinese fir forests in the study area; Table S2. Original data on stand biomass and carbon stocks of 65 plots of Chinese fir forests in the study area.
