# Supplementary material for: Regional Variation in Vegetation Biomass and CSDLv2-Derived Soil C:N:P Stoichiometry Across Chinese Fir Plantations of Different Ages: An Exploratory Comparison
Source: Plants (Basel). 2026 Oct 22;15(21):3245. doi: 10.3390/plants15213245 (PMC13766173; doi:10.3390/plants15213245)
Supplement: Supplementary file 1 [file plants-15-03245-s001.zip › plants-4592352-supplementary.pdf]

Table S1. Original basic information of 65 plots of Chinese fir forests in the study area

Plot ID	Latitude	Longitude	Stand age (yr)	Developmental stage	Plot area (ha)
1	26.881	113.013	7	Young	0.0667
2	26.452	109.357	7	Young	0.0667
3	26.681	111.076	7	Young	0.0667
4	26.677	111.079	7	Young	0.0667
5	27.461	113.057	8	Young	0.0667
6	27.354	112.303	8	Young	0.0667
7	28.981	110.493	8	Young	0.0667
8	26.053	109.481	9	Young	0.0667
9	26.168	109.840	9	Young	0.0667
10	25.323	112.021	10	Middle-Age	0.0667
11	24.871	111.594	10	Middle-Age	0.0667
12	28.285	109.771	10	Middle-Age	0.0667
13	27.212	109.547	10	Middle-Age	0.0667
14	25.991	111.800	11	Middle-Age	0.0667
15	26.709	109.794	11	Middle-Age	0.0667
16	28.131	110.059	12	Middle-Age	0.0667
17	26.273	111.938	12	Middle-Age	0.0667
18	28.014	110.837	13	Middle-Age	0.0667
19	25.737	111.758	14	Middle-Age	0.0667
20	27.544	110.555	14	Middle-Age	0.0667
21	28.265	110.552	14	Middle-Age	0.0667
22	25.820	113.776	15	Middle-Age	0.0667
23	27.825	112.503	15	Middle-Age	0.0667
24	27.722	110.108	15	Middle-Age	0.0667
25	26.477	113.857	16	Middle-Age	0.0667
26	26.457	109.757	16	Middle-Age	0.0667
27	27.250	112.252	17	Middle-Age	0.0667
28	28.568	113.535	18	Middle-Age	0.0667
29	28.760	109.618	18	Middle-Age	0.0667
30	25.907	112.917	18	Middle-Age	0.0667
31	27.981	110.048	18	Middle-Age	0.0667
32	28.269	114.017	19	Middle-Age	0.0667
33	25.053	112.033	21	Near-Mature	0.0667
34	27.363	110.919	21	Near-Mature	0.0667
35	28.843	110.385	21	Near-Mature	0.0667
36	24.979	111.872	22	Near-Mature	0.0667
37	27.216	109.951	22	Near-Mature	0.0667
38	28.471	112.417	22	Near-Mature	0.0667
39	27.506	110.272	22	Near-Mature	0.0667
40	25.923	114.004	23	Near-Mature	0.0667
41	26.544	110.979	23	Near-Mature	0.0667
42	29.002	110.520	24	Near-Mature	0.0667

43	28.517	110.183	24	Near-Mature	0.0667
44	27.388	112.820	25	Mature	0.0667
45	25.390	111.956	25	Mature	0.0667
46	28.007	109.617	25	Mature	0.0667
47	27.095	112.483	25	Mature	0.0667
48	26.924	109.631	25	Mature	0.0667
49	27.400	110.798	25	Mature	0.0667
50	25.894	113.714	26	Mature	0.0667
51	25.207	112.123	27	Mature	0.0667
52	27.651	110.433	28	Mature	0.0667
53	27.471	110.880	29	Mature	0.0667
54	25.914	109.723	31	Over-Mature	0.0667
55	25.336	112.272	32	Over-Mature	0.0667
56	25.122	112.032	32	Over-Mature	0.0667
57	29.000	110.520	33	Over-Mature	0.0667
58	29.575	113.484	34	Over-Mature	0.0667
59	26.930	110.436	34	Over-Mature	0.0667
60	28.006	112.465	35	Over-Mature	0.0667
61	28.662	111.574	35	Over-Mature	0.0667
62	27.410	113.056	40	Over-Mature	0.0667
63	26.243	110.320	42	Over-Mature	0.0667
64	26.158	113.041	44	Over-Mature	0.0667
65	27.338	110.429	45	Over-Mature	0.0667

Table S2. Original data on stand biomass and carbon stocks of 65 plots of Chinese fir forests in the study area

Plot ID	Aboveground biomass	Belowground biomass	Total biomass	Aboveground C stock	Belowground C stock	Total C stock
1	2970.402	665.208	3635.610	1421.635	293.224	1714.858
2	906.432	201.962	1108.394	433.819	89.025	522.843
3	407.805	90.646	498.451	195.176	39.957	235.132
4	403.987	89.077	493.064	193.348	39.265	232.613
5	1747.476	390.453	2137.929	836.342	172.112	1008.454
6	714.407	158.025	872.432	341.915	69.657	411.573
7	6548.101	1514.551	8062.653	3133.921	667.614	3801.536
8	126.083	28.036	154.119	60.343	12.358	72.702
9	4452.800	1003.795	5456.595	2131.110	442.473	2573.583
10	1678.074	375.544	2053.618	803.126	165.540	968.666
11	721.640	159.058	880.699	345.377	70.113	415.490
12	4648.122	1046.896	5695.017	2224.591	461.472	2686.063
13	1778.020	399.155	2177.176	850.960	175.948	1026.908
14	2714.991	610.886	3325.877	1299.395	269.279	1568.673
15	3431.001	768.226	4199.227	1642.077	338.634	1980.711
16	5496.835	1240.402	6737.236	2630.785	546.769	3177.554
17	6648.292	1503.949	8152.241	3181.873	662.941	3844.813
18	7530.971	1695.420	9226.391	3604.323	747.341	4351.664
19	841.008	188.681	1029.688	402.506	83.170	485.677
20	4903.744	1108.772	6012.516	2346.932	488.747	2835.678
21	2022.933	447.884	2470.817	968.176	197.427	1165.603
22	6241.625	1397.712	7639.337	2987.242	616.111	3603.353
23	3232.440	737.145	3969.585	1547.046	324.933	1871.979
24	3691.584	835.506	4527.090	1766.792	368.291	2135.083
25	3616.416	822.825	4439.241	1730.817	362.701	2093.518
26	6554.324	1483.794	8038.118	3136.900	654.056	3790.956
27	2835.659	637.811	3473.470	1357.146	281.147	1638.293
28	7578.469	1716.576	9295.045	3627.055	756.667	4383.722
29	5649.456	1276.913	6926.369	2703.830	562.863	3266.693
30	2053.151	462.340	2515.492	982.638	203.800	1186.438
31	1944.952	440.057	2385.009	930.854	193.977	1124.831
32	2111.967	470.388	2582.355	1010.788	207.347	1218.135
33	3941.940	892.323	4834.263	1886.612	393.336	2279.948
34	1385.722	311.851	1697.573	663.206	137.464	800.670
35	5129.726	1180.528	6310.253	2455.087	520.377	2975.463
36	4659.943	1064.375	5724.318	2230.249	469.177	2699.425
37	2281.572	516.794	2798.366	1091.960	227.803	1319.763
38	4084.039	923.308	5007.348	1954.621	406.994	2361.616
39	2084.851	476.948	2561.799	997.810	210.239	1208.048
40	1387.620	311.064	1698.684	664.115	137.117	801.232
41	6002.836	1359.360	7362.196	2872.957	599.206	3472.163
42	5432.249	1250.684	6682.933	2599.874	551.301	3151.176
43	1923.349	433.224	2356.572	920.515	190.965	1111.480
44	8017.297	1837.613	9854.911	3837.079	810.020	4647.098
45	3777.664	874.759	4652.423	1807.990	385.594	2193.584

46	4811.945	1099.314	5911.258	2302.997	484.577	2787.574
47	3340.471	764.031	4104.502	1598.750	336.785	1935.534
48	4755.538	1083.778	5839.316	2276.001	477.729	2753.730
49	4323.430	990.850	5314.280	2069.194	436.767	2505.960
50	1077.498	242.224	1319.722	515.690	106.772	622.463
51	13131.399	3009.981	16141.380	6284.688	1326.800	7611.487
52	3293.492	748.322	4041.813	1576.265	329.860	1906.125
53	4233.047	971.320	5204.367	2025.936	428.158	2454.094
54	5240.593	1209.748	6450.341	2508.148	533.257	3041.405
55	8857.181	2032.119	10889.301	4239.047	895.758	5134.805
56	7587.606	1728.485	9316.090	3631.428	761.916	4393.344
57	4954.603	1152.387	6106.991	2371.273	507.972	2879.245
58	4990.524	1134.891	6125.415	2388.465	500.260	2888.725
59	2920.293	665.269	3585.562	1397.652	293.251	1690.903
60	4975.384	1125.965	6101.349	2381.219	496.325	2877.544
61	1638.324	370.804	2009.128	784.102	163.451	947.552
62	3229.314	740.657	3969.971	1545.550	326.482	1872.031
63	5834.195	1347.161	7181.356	2792.246	593.828	3386.074
64	4261.525	972.127	5233.652	2039.566	428.513	2468.079
65	5186.568	1199.019	6385.587	2482.291	528.528	3010.819
